# Short-term ambient PM_2.5_ exposure and cause-specific mortality in Massachusetts: Effect modification by structural air exchange rates

**DOI:** 10.1097/EE9.0000000000000385

**Published:** 2025-04-16

**Authors:** Futu Chen, Jaime E. Hart, Jarvis T. Chen, Brent A. Coull, M. Patricia Fabian, Joel Schwartz, Gary Adamkiewicz

**Affiliations:** a Department of Environmental Health, Harvard T.H. Chan School of Public Health, Boston, Massachusetts; b Channing Division of Network Medicine, Department of Medicine, Brigham and Hospital and Harvard Medical School, Boston, Massachusetts; c Department of Social and Behavioral Sciences, Harvard T. H. Chan School of Public Health, Boston, Massachusetts; d Department of Biostatistics, Harvard T. H. Chan School of Public Health, Boston, Massachusetts; e Department of Environmental Health, Boston University School of Public Health, Boston, Massachusetts; f Institute for Global Sustainability, Boston University, Boston, Massachusetts

**Keywords:** Air exchange rate, effect heterogeneity, Ambient air pollution, Exposure measurement, Building, Mortality

## Abstract

**Background:**

Air exchange rate (AER) is a ventilation factor determining the infiltration of ambient air pollution indoors. AER has been under-examined as a source of air pollution health effect heterogeneity. Therefore, we assessed parcel-level structural AER as a modifier of the association between short-term ambient PM_2.5_ exposure and cause-specific mortality in Massachusetts.

**Methods:**

In this time-stratified case-crossover study, we included 770,836 nonaccidental deaths between 5 January 2000 and 31 December 2015. We examined the association between short-term ambient PM_2.5_ and risk of cause-specific death with conditional logistic regression. Effect modification by seasonal parcel-level AER was examined with a season-specific AER and PM_2.5_ product term, and stratification on parcel housing and residential type.

**Results:**

PM_2.5_ in the preceding 2 days (lag 0–1) was associated with overall and cardiovascular mortality. Effect modification by AER was only observed in the warm season for all-cause and respiratory mortality. During the warm season among multifamily parcels, we observed the strongest effect modification by AER for respiratory mortality (odds ratio of interaction term: 1.15, 95% confidence interval [CI]: 1.05, 1.27). This corresponds to effect estimates per a 10 μg/m^3^ increase in PM_2.5_ at the 25th percentile of AER (0.27/hour) that is associated with a 0.3% decrease in respiratory mortality (95% CI: −5%, 4%), and an 8.2% increase of respiratory mortality at the 75th percentile of AER (0.85/hour) (95% CI: 2%, 15%).

**Conclusion:**

Parcel-level AER was a modifier of the association between short-term ambient PM_2.5_- and mortality during warm seasons. Parcel-level AER may help us to better quantify associations between pollution and health outcomes.

**What this study adds:**

This is the first study to use fine spatial resolution (parcel-level) structural air exchange rates (AERs) to study the effect of heterogeneity due to ventilation factors in air pollution epidemiology. We used a case-crossover study design and estimated effect modification of the association between short-term ambient PM_2.5_ exposure and cause-specific mortality in Massachusetts by AERs. We found that structural AER was a modifier during warm seasons. This work implies that including parcel-level AER may help us better quantify ambient pollution associations.

## Introduction

Acute exposure to ambient fine particulate matter with a diameter of less than 2.5 µm (PM_2.5_) has been consistently associated with increased all-cause, cardiovascular (CVD), and respiratory mortality at local and national scales.^1,2^ Most epidemiological studies have measured exposure as ambient concentrations either from monitoring sites (e.g., Olstrup et al^3^) or model predictions (e.g., Di et al^4^; Kloog et al^5^; Wei et al^6^). With people spending most of their time indoors,^7^ however, there may be a discrepancy between ambient measures and personal exposure to ambient-sourced pollutants that can contribute to measurement error in epidemiologic studies that utilize ambient measures as a proxy for personal exposure. If not addressed adequately, exposure measurement error (non-pure Berkson error) would contribute to a downward bias in effect estimates, inflated standard errors, and wider confidence intervals (CIs) if the error is nondifferential.^8,9^ More seriously, if the measurement error in the exposure is dependent on additional factors, for example, an effect modifier of interest, then such differential measurement error would additionally impair our ability to identify effect measure modifications in susceptible populations.^10^

Air exchange rate (AER) is a building ventilation factor that contributes to the infiltration of ambient air into indoor spaces and is, thus, one of the determinants of residential air pollution.^11^ Physical characteristics such as building age and building constructions that determine AERs vary socioeconomically and demographically.^12,13^ In addition, poor physical conditions are correlated with excessively higher environmental exposures and thereby increase the health risks for populations that live in those buildings.^14^ Rosofsky et al^15^ used a model developed by a team at Lawrence Berkeley National Labs^16^ to develop parcel-level AER estimates for the Commonwealth of Massachusetts (MA). They demonstrated that there was more variation between parcel-level AERs than the variation between 1 km × 1 km gridded ambient PM_2.5_.^15^ In addition, if parcel-level AER were used to modify ambient exposures, higher ambient-sourced PM_2.5_ exposures were observed among block groups that have a higher percentage of minority populations, with lower educational attainments and with lower median home values.^15^

AER has been applied in epidemiological studies to quantify variability in residential exposures. For instance, Sarnat et al^13^ estimated zip-code level structural AER in Atlanta as an effect modifier to study air pollution-related acute asthma emergency department visits. Hodas et al^17^ explored effect modification by census-tract level structural AERs for ambient PM_2.5_ exposure and transmural myocardial infarction incidence in New Jersey. One of the limitations of these studies is that AERs were based on zip-code or census-tract level estimates^13,17^; therefore, the authors suggested the need for a more detailed AER model (comparison of different spatial units expanded in Figure S.1; https://links.lww.com/EE/A339).

While previous works explored the extent to which AER modifies PM_2.5_ associations with morbidity, there is little work related to mortality. We believe that incorporating it is important to assess its impact on air pollution and mortality associations. This article includes parcel-level AER estimates to investigate the potential for effect modification of the association between short-term ambient PM_2.5_ exposure and cause-specific mortality in MA from 2000 to 2015.

## Methods

### Study population and unit of analysis

The study population consisted of 770,836 nonaccidental deaths to those aged 40 years or older who resided in Massachusetts, with an identifiable International Classification of Disease (ICD) code between 5 January 2000 and 31 December 2015. Death records were obtained from the Massachusetts Department of Public Health death registry, where each death record included geocoded residential address, age at death, sex, race/ethnicity, education, occupation, date of death, and underlying cause of death with its corresponding ICD, 10th Revision (ICD-10) code. In addition to all-cause mortality, we identified CVD deaths (n = 258,245 ICD-I codes) and respiratory deaths (n = 85,876, ICD-J codes as well as C030-035 and C045).

The geocode associated with each death was matched to its nearest parcel published by the Metropolitan Area Planning Council (MAPC).^18^ Deaths matched to a nonresidential parcel belonging to agriculture and outdoor recreational activities, educational uses, or industrial properties, warehouse and utilities (n = 16,027, 2.08%), or has a building area of 0 (n = 40,215, 5.22%) were excluded. We further excluded deaths greater than 10 m away from a parcel (n = 8,748, 1.13%), resulting in analytical n of 707,836 all-cause mortality, among them, 236,320 CVD deaths and 78,529 respiratory deaths. Details of this exclusion process are included in Figure S.2; https://links.lww.com/EE/A339. Exclusion criteria were based on matching precision and the quality of parcel AER estimates (expanded in Supplemental methods; https://links.lww.com/EE/A339).

This study was approved by the Harvard T.H. Chan School of Public Health Human Subjects Committee.

### Exposure and covariates

Ambient average daily PM_2.5_ from 2000 to 2015 was estimated from a previously published spatial model.^19^ Briefly, the authors used an ensemble approach that combined three machine learning algorithms on predictors such as PM_2.5_ monitoring data (24 hours average), satellite data, meteorological variables, and land-use variables to estimate daily mean ambient PM_2.5_ concentration at a 1 km × 1 km spatial resolution for entire contiguous United States. Missing predictor variables were interpolated with a random forest or simple linear interpolation.^19^ Cross-validation indicated a good model performance (10-fold cross-validated R^2^ for daily values of 0.89 in the New England region). Details of quality assurance processes are described in Di et al.^19^

Ambient average daily temperature, relative humidity, and day of the week were included as potential confounders. Daily minimum and maximum temperature (in °C), as well as relative humidity (%) were obtained for the closest grid centroid of the gridded meteorological dataset at a 4 km × 4 km spatial resolution.^20^ We averaged daily minimum and maximum temperatures as an approximation to the daily mean.

### Effect modifiers

We estimated structural AER at the parcel level for all residential and nonresidential parcels in Massachusetts during warm and cool seasons. Residential parcels were identified by MAPC real estate type 1 to 5: type 1 = single family properties; type 2 = duplex/triplex; type 3 = small apartments; type 4 = large apartments; and type 5 = multiuse residential properties.^18^ Nonresidential living parcels were classified through the following criteria: (1) not a road, driveway, open area, or water feature; (2) not defined as agriculture and outdoor recreational activities, educational uses such as universities, and industrial properties, warehouse, and utilities; (3) contained a geocode associated with a death(s) between 2000 and 2015.

Methods for calculating the parcel AERs have been published elsewhere^15^ and details are included in Supplemental methods; https://links.lww.com/EE/A339.

In brief, AER predictions were based on the Lawrence Berkeley National Laboratory physical-based infiltration model, which predicts structural parcel leakage rates through physical mechanisms.^16^ The model integrates the year built, the number of stories, building area (m^2^), indoor temperature, land use, seasonal wind speed, and seasonal ambient temperature. Natural ventilation, such as window opening, or mechanical ventilation, such as heating, ventilation, and air conditioning (HVAC) systems, are not considered. Rosofsky et al^15^ expanded the modified AER equation from single-family exclusively to single-family and multifamily residential homes. In this work, we expanded the estimation to nonresidential parcels in MA (see Table S.4 and Supplemental methods; https://links.lww.com/EE/A339). Figure S.3 and Table S.2. (https://links.lww.com/EE/A339) illustrate the differences in AER between residential and non-residential parcels.

We also assessed the type of the parcel and parcel housing types as potential effect modifiers. The type of parcel, residential versus nonresidential, was defined by MAPC real estate type.^18^ The parcel-level housing types, single-family versus multifamily, were defined by MAPC real estate type among residential parcels.^18^ Certain parcels, such as nursing homes and buildings owned by city government, were classified as nonresidential. Therefore, within nonresidential parcels, we defined single-family parcels as those with less than two estimated housing units. We treated all identifiable nonresidential affordable housing parcels as multifamily parcels (Supplemental methods; https://links.lww.com/EE/A339).

## Statistical methods

We assessed the association between short-term ambient PM_2.5_ and cause-specific mortality using the time-stratified case-crossover method.^21^ A case-crossover design mimics a case-control approach, but with “control days” on which the event did not occur selected to be at the same address of the decedent, and close to the “case day” on which an event occurred. For example, in this analysis, for each individual, the case day was the date of the death, and control days were every third day before or after the case day in the same month and the same year (roughly 1:9 case:control matching). For both case and control days, ambient PM_2.5_ exposure was defined as the moving average of current and previous day concentrations (0–1 lag). We selected this exposure window based on previous evidence^22,23^ and sensitivity analyses across different exposure windows (Table S.3.; https://links.lww.com/EE/A339). Since the data are matched by individual, confounding by individual time-invariant or slowly varying characteristics are effectively controlled by matching. We controlled for potential confounding by time-varying factors by entering daily mean temperature, daily relative humidity, and day of the week in the model.

Model parameter estimates were obtained via conditional logistic regression using the R package “survival.”^24,25^ The model specification is as follows:

$$\begin{array}{l} \log\left(\frac{P_{ij}}{1-p_{ij}}\right)=\alpha_{i}+\beta_{1}PM2.5_{ij}+C_{ij}^{t}\gamma , \end{array}$$ (Eq.1)

with p denoting mortality risk from ambient PM_2.5_ exposure, i denoting stratum (a matched set), and j denoting day within stratum. Confounders are listed as a vector C.

The linearity of the main effects was checked by fitting the conditional logistic regression with a linear term for ambient PM_2.5_ exposure and a smooth term without penalty to the null space and checking whether the smooth term was significant at the alpha = 0.05 significance level.^26^

To examine effect modification by seasonal AERs, we stratified case days by warm season (May to October) and cool season (November to April). We first checked the linearity of effect modification (assuming a linear ambient PM_2.5_ effect) in each model by adding a smooth term of AER by PM_2.5_ and assessed whether the smooth term was significant at the prespecified 0.05 level. We then tested multiplicative effect modification by adding an AER and PM_2.5_ product term to the conditional logistic regression model. To account for effect modification through other building characteristics, models were further stratified by residential types and housing types.

Effect modification was examined by odds ratios (ORs) that were associated with the product terms and their 95% CIs. These ORs correspond to effect estimates per 10 µg/m³ increase in ambient PM_2.5_, per unit (hour^−1^) increase in AER. For interpretability, we reported this effect modification by % increase in the risk of cause-specific death in each season and their 95% CI at 25% and 75% percentiles of AER.

To account for potential air-conditioning use during the warm season that might affect parcel AER estimates, we performed sensitivity analyses when the ambient temperature of the death day is <29°C. To account for death that might not occur at the residential address, additional sensitivity analyses were performed excluding both in-patient and outpatient in-hospital death.

Parcel AERs were estimated using SAS 9.3 (SAS Institute Inc., Cary, NC). Remaining statistical analysis was carried out in R 3.6.1. with package “mgcv” and “survival.”^24,27^

## Results

### Descriptive characteristics of the study population, exposure, and effect modifiers

Table 1 describes the characteristics of the cases. Among total nonaccidental mortality cases, 8.17% were located greater than 10 m from a residential or nonresidential parcel boundary or in a nonresidential parcel with zero building area. After excluding those cases, we included 707,836 all-cause mortality cases. Of them, 33% (N = 236,320) were CVD mortality, and 11% (N = 78,529) were respiratory mortality.

**Table 1.** Characteristics of the study population (Massachusetts Department of Public Health death registry) and the distribution of exposure, confounder, and effect modifiers in the study population by cause-specific mortalities in Massachusetts, USA, 2000 to 2015

	All-cause mortality (N = 707,836)	CVD mortality (N = 236,320)	Respiratory mortality (N = 78,529)
Sex			
Male	325,433 (46.0%)	108,846 (46.1%)	35,300 (45.0%)
Female	382,389 (54.0%)	127,468 (53.9%)	43,229 (55.0%)
Missing	14 (0.0%)	6 (0.0%)	0 (0%)
Age (years)			
Mean (SD)	78.1 (13.1)	80.4 (12.4)	80.4 (11.0)
Median (min, max)	81.0 (40.0, 117)	83.0 (40.0, 115)	82.0 (40.0, 110)
Race			
White	660,897 (93.4%)	222,005 (93.9%)	75,091 (95.6%)
Black	27,330 (3.9%)	8,741 (3.7%)	1,802 (2.3%)
Other	19,534 (2.8%)	5,547 (2.3%)	1,631 (2.1%)
Missing	75 (0.0%)	27 (0.0%)	5 (0.0%)
Education			
Less than high school	123,636 (17.5%)	44,109 (18.7%)	15,343 (19.5%)
High School	366,887 (51.8%)	123,153 (52.1%)	42,127 (53.6%)
More than high school	209,171 (29.6%)	66,091 (28.0%)	19,938 (25.4%)
Missing	8,142 (1.2%)	2,967 (1.3%)	1,121 (1.4%)
PM_2.5_ (µg/m³, lag 0–1)			
Mean (SD)	8.90 (5.19)	8.99 (5.26)	8.93 (5.18)
Median (min, max)	7.60 (0.0232, 62.8)	7.67 (0.102, 60.5)	7.64 (0.101, 62.8)
IQR (Q1, Q3)	5.77 (5.33, 11.1)	5.86 (5.37, 11.2)	5.77 (5.37, 11.1)
Temperature (°C, lag 0–1)			
Mean (SD)	9.43 (9.71)	9.29 (9.70)	8.74 (9.73)
Median (min, max)	9.40 (−21.2, 31.0)	9.18 (−20.5, 31.0)	8.28 (−21.2, 30.5)
IQR (Q1, Q3)	16.3 (1.65, 17.9)	16.3 (1.48, 17.8)	16.1 (1.08, 17.2)
RH (%, lag 0–1)			
Mean (SD)	64.5 (9.99)	64.5 (9.97)	64.1 (10.1)
Median (min, max)	65.5 (27.1, 100)	65.6 (27.6, 100)	65.2 (27.3, 100)
IQR (Q1, Q3)	13.3 (58.3, 71.6)	13.3 (58.3, 71.6)	13.6 (57.8, 71.3)
Parcel residential type			
Nonresidential	195,533 (27.6%)	66,860 (28.3%)	24,136 (30.7%)
Residential	512,303 (72.4%)	169,460 (71.7%)	54,393 (69.3%)
Parcel housing type			
Single-family	432,569 (61.1%)	144,063 (61.0%)	47,210 (60.1%)
Multifamily	275,267 (38.9%)	92,257 (39.0%)	31,319 (39.9%)
Seasonal AER (/hour)			
Mean (SD)	0.629 (0.495)	0.632 (0.497)	0.631 (0.496)
Median (min, max)	0.513 (0.00, 6.06)	0.516 (0.00, 6.06)	0.519 (0.00, 5.64)
IQR (Q1, Q3)	0.584 (0.270, 0.854)	0.587 (0.272, 0.859)	0.594 (0.268, 0.862)

IQR indicates interquartile range; Q1, 25 percentile; Q3, 75 percentile; SD, standard deviation.

The mean age at death was 78 (±SD = 13) years, 45% to 46% were females, the majority were white (including Hispanic Whites, 94%), and had a high school education or less (Table 1). Overall, 72.4% of the cases lived in residential parcels, and 61.1% lived in single-family parcels. The mean 0–1 lag average PM_2.5_ level was approximately 8.9 µg/m³ with an interquartile range (IQR) of approximately 5.8 µg/m³.

During the cool season, 219,856 unique parcels contained a death; during the warm season, cases were joined into 201,062 unique parcels. Overall, AER had a mean of 0.63/hour (SD = 0.50/hour) with an IQR of 0.58/hour. Mean and median of cool season AER was higher than the warm season and had wider IQRs (Table 2). Mean and median AER among single-family parcels were lower than multifamily parcels, and compared with residential parcels, AER was lower among nonresidential parcels (Table 2). Figure S.4 (https://links.lww.com/EE/A339) presents the spatial distribution of census-tract level crude all-cause mortality rate included in this study in 2010 and AER averaged at census-tract level.

**Table 2.** Seasonal parcel-level air exchange rate in Massachusetts, stratified by housing and residential type

Seasonal AER	Cool season				Warm season			
	Housing type		Residential type		Housing type		Residential type	
	Single-family (N = 229,054)	Multifamily (N = 145,395)	Residential (N = 270,206)	Nonresidential (N = 104,243)	Single-family (N = 203,515)	Multifamily (N = 129,872)	Residential (N = 242,097)	Nonresidential (N = 91,290)
Mean (SD)	0.72 (0.44)	1.00 (0.62)	0.94 (0.52)	0.55 (0.47)	0.38 (0.28)	0.44 (0.37)	0.48 (0.32)	0.18 (0.17)
Median (min, max)	0.68 (0.00, 5.64)	0.95 (0.00, 6.06)	0.83 (0.02, 6.06)	0.43 (0.00, 5.23)	0.33 (0.00, 3.99)	0.34 (0.00, 4.45)	0.41 (0.006, 4.45)	0.14 (0.00, 3.84)
IQR (Q1, Q3)	0.52 (0.43, 0.94)	0.89 (0.51, 1.40)	0.64 (0.57, 1.21)	0.55 (0.20, 0.75)	0.30 (0.19, 0.50)	0.43 (0.17, 0.60)	0.34 (0.27, 0.61)	0.19 (0.06, 0.25)

IQR indicates Interquartile range; Q1, 25 percentile; Q3, 75 percentile; SD, standard deviation.

### Effect of short-term ambient PM_2.5_ and cause-specific mortality

Each 10 µg/m³ increase in ambient present and previous day average PM_2.5_ was statistically significantly associated with a 2.1% (95% CI: 1.5%, 2.7%) increase in all-cause mortality and 2.0% (95% CI: 1.0%, 3.0%) increase in CVD mortality adjusting for the day of the week, ambient daily average temperature and ambient daily average relative humidity (Table 3). We did not find evidence of a nonlinear effect of PM_2.5_ in each cause-specific death in each season (*P*-value for smooth function >0.05; Table S.4; https://links.lww.com/EE/A339).

**Table 3.** Adjusted odds ratios of death and 95% CIs from conditional logistic regressions by cause-specific mortality during the study period (2000 to 2015), Massachusetts, USA

Predictors	All-cause mortality N = 7,160,723		CVD mortality N = 2,390,165		Respiratory mortality N = 793,618	
	Odds ratio (95% CI)	*P*-value	Odds ratio (95% CI)	*P*-value	Odds ratio (95% CI)	*P*-value
PM_2.5_ (10 µg/m^3^ increase)	1.02 (1.01, 1.03)	**<0.001**	1.02 (1.01, 1.03)	**<0.001**	1.01 (0.99, 1.03)	0.263
Temperature (10°C increase)	1.00 (1.00, 1.01)	0.268	0.99 (0.98, 1.01)	0.254	1.01 (0.99, 1.03)	0.418
RH (10% increase)	1.00 (1.00, 1.01)	0.061	1.01 (1.00, 1.01)	**0.038**	1.00 (0.99, 1.01)	0.818

Models also adjusted for day of the week (results not shown). Boldfaced value indicates significant coefficients (i.e., *P*-value greater than 0.05).

CI indicates confidence interval; RH, relative humidity.

### Effect modification by structural AER

**Figure 1.**
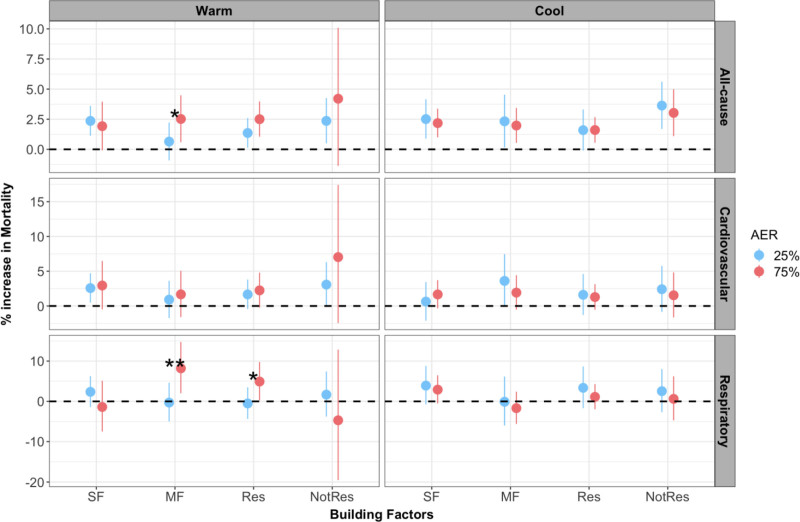
Estimated percentage change in cause-specific mortality associated with a 10µg/m^3^ increase in PM_2.5_ at 15% and 75% AER, stratified by season, parcel housing, and residential type, 2000 to 2015, Massachusetts, USA. Significance for the PM_2.5_*AER product term on the multiplicative scale: *P < 0.05; **P < 0.01. MF, multifamily; NotRes, nonresidential parcels; Res, residential; SF, single family.

We did not observe a nonlinear effect modification of AER upon the linear effect of PM_2.5_ (*P*-value for smooth function >0.05; Table S.5; https://links.lww.com/EE/A339). Higher AER was associated with an increase in PM_2.5_-associated mortality during the warm season without stratification (Table S.6; https://links.lww.com/EE/A339). The pattern was not consistent during the cool season. Such seasonal differences persisted after further stratification by housing and residential types (Figure 1 and Table S.7; https://links.lww.com/EE/A339). We observed significant effect modification by AER of the PM_2.5_ effect during the warm season among multifamily and residential parcels for all-cause and respiratory mortalities. However, AER did not modify the short-term ambient PM_2.5_ and mortality association during the cool season (Figure 1).

During the warm season, the strongest effect modification by AER was observed among people living in multifamily parcels and for respiratory mortality (OR of interaction term: 1.15, 95% CI: 1.05, 1.27). This corresponds to effect estimates per a 10 μg/m^3^ increase in PM_2.5_ at the 25th percentile of AER (0.27/hour) that is associated with a 0.3% decrease in respiratory mortality (95% CI: −5%, 4%), and an 8.2% increase of respiratory mortality at the 75th percentile of AER (0.85/hour) (95% CI: 2%, 15%). The significant effect modification was weaker for all causes of death located at multifamily parcels (OR of interaction term: 1.03, 95% CI: 1.00, 1.06) but consistent in direction. This modification corresponds to an increase of PM_2.5_-related all-cause mortality by 2.52% (95% CI: 0.59%, 4.49%) for parcels at the 75th percentile of AER, as opposed to a 0.64% increase (95% CI: −0.91%, 2.22%) in parcels at the 25th percentile of AER, among multifamily parcels.

Figure 1 also illustrates the pattern of effect modification, where during the warm season, despite not seeing a significant modification among CVD deaths, higher risks were observed at higher AER parcels. Stratification by parcel residential type only revealed significant effect modification during the warm season among respiratory mortality in residential parcels (OR of product term = 1.10, 95% CI: 1.01, 1.19; Figure 1).

In sensitivity analyses, we did not find evidence of a nonlinear effect of PM_2.5_ or the PM_2.5_ and AER interaction term (Table S.4; https://links.lww.com/EE/A339, Table S.5; https://links.lww.com/EE/A339). We observed consistent results during warm season when ambient temperature was <29 °C (Table S.8; https://links.lww.com/EE/A339). After excluding 39.9% and 41.0% in-hospital death during warm season and cool season, respectively, results showed similar effect modifications by parcel AER with stronger magnitude (Table S.9; https://links.lww.com/EE/A339, and Figure S.5; https://links.lww.com/EE/A339).

## Discussion

In this study with 16 years of death data, we found that exposure to short-term ambient PM_2.5_ was associated with increased all-cause and CVD mortality. This finding is well aligned with previous state-wide and national-wide studies.^5,6,22,23^ Unlike previous studies, we did not observe associations with respiratory mortality; however, previous studies also suggested that the effects of ambient particles on respiratory mortality were strongest with longer exposure windows or very high exposures.^28–30^

The current study also investigated the effect modification of the PM_2.5_ effect by parcel-level structural AER estimates. Effect modification by AER was only observed during the warm season among multifamily parcels for all-cause and respiratory mortality. Relative risks associated with higher PM_2.5_ exposure were higher at high AER compared to lower AER, as expected. Sarnat et al^31^ illustrated that the association between ambient PM_2.5_ concentration and personal ambient-sourced PM_2.5_ measures was the strongest among well-ventilated senior citizen’s homes, indicating that higher ventilation (as represented by AER in this study) increases the infiltration of ambient exposure. In a pooled multicity analysis, Baxter et al^32^ reported that the city-wide effect between ambient PM_2.5_ concentrations and nonaccidental all-cause mortality was higher among cities with older and larger homes with less air-conditioning. These studies demonstrated that higher ventilation was associated with a higher ambient exposure-mortality risk.

Few studies have explored AER as an effect modifier for PM-associated mortality and morbidity; of them, similar findings were reported among myocardial infarction patients during warm months and hospital ED visits.^13,17^ Significant findings were reported when AER was treated as terciles or combined middle and high categories.^13,17^ Given that we did not find evidence of nonlinear effect modification, we did not categorize parcel AER in this study. Still, our findings were in line with previous publications. In addition, with AER data with finer geographical resolution, we were able to further stratify by housing or building factors that not only regulate AER but also had a socioeconomic pattern. The review by Dionisio et al^33^ suggested that in the case-crossover design, stratification by key household factors is critical to detect effect modification by AER. In our study population, multifamily parcels were more likely to be located in census tracts with a higher %non-White population and higher prevalence of living below the poverty level. Significant results among multifamily parcels indicated that population in certain neighborhoods (census tracts) are at higher risk for ambient-sourced PM_2.5_ exposure due to proximity to ambient pollution sources, and housing and building factors.

In our analysis, while the main effects of PM_2.5_ were not significant among respiratory deaths, we observed significant effect modification of these effects by AER for this outcome, with interaction estimates larger than those for all-cause mortalities. In a study with 27 US communities, researchers also reported that certain housing characteristics, such as central air-conditioning explained 60% of the estimated city variation of ambient PM_2.5_-associated respiratory deaths.^34^ Although central air-conditioning was not used in the estimation of structural AER, their finding hinted that ventilation as well as socioeconomic status might contribute to effect variation at the city level. The present study echoed this result and confirmed substantial effect heterogeneity for respiratory deaths at the parcel level.

We saw mixed patterns of effect modification during the cool season, which can possibly be attributed to the seasonal PM_2.5_ distribution. In our study, the warm season PM_2.5_ was higher than the cool season. During cool season, exposure to ambient-sourced exposures might not be as comparable as warm season. Also, during the cool season, the pattern of spending more time indoors where there is air filtration by building envelope may overwhelm the variation in that filtration.

This study has several limitations. First, some of our geocoded death data were not located directly inside a parcel. The nearest neighbor method reduced the loss of data but also increased the plausibility of spatial mismatch. To improve the precision, we excluded deaths that were too far away (>10 m) from a parcel. After exclusion, the median distance between death and a parcel boundary was <0.01 m with IQR ≤ 0.01 m (mean = 0.54 m), indicating a good match. Another limitation of the spatial join is the potential for temporal mismatch created between time-invariant measures of parcel characteristics and deaths, which are time-specific. Mismatches may exist if the parcel characteristics change over time. Additional mismatches could occur if patients passed away in the hospital during the exposure window. Therefore, in sensitivity analyses, we excluded in-hospital deaths and arrived at similar findings.

Second, there are uncertainties in our AER estimations. We are unable to validate the modeled AER against measured AER. However, the previous study has found that using the Lawrence Berkeley National Laboratory model is a close approximation of measured AER, assuming windows are closed.^15^ Another uncertainty is that AER was estimated through a physical model that only provides insight into structural AER (AER due to building leakage).^35^ We did not consider behavioral factors such as window opening and mechanical ventilation such as HVAC systems that would increase or decrease the residential AERs.^36,37^ These factors are harder to model and not available in public datasets. However, since structural AER, as a building characteristic, is a rough baseline of residential AER, assuming that these behavior and mechanical factors are nondifferential and that higher AER is associated with higher PM_2.5_-related mortality, our structural AER would be an underestimation of true AER, particularly in the summer. Finally, we had no information on indoor sources of PM_2.5_ such as gas stoves or smoking, contributing to the exposure measurement error by using ambient exposure as a proxy for personal exposure. However, we believe that the measurement error is nondifferential, which may attenuate the observed association, after controlling for the short-term temporal changes. AER also impacts indoor-sourced PM_2.5_ moving indoor air pollutants to outdoors. Our study design (case-crossover) does not explore the net effect of AER on mortality. Instead, our study question focuses on the effect modification of AER on ambient-sourced PM_2.5_. Therefore, the interpretation of our results should only be restricted to ambient-sourced PM_2.5_.

The biggest strength of our study is its use of the high spatial resolution of structural AER estimates and the long study period. Compared with the previous evidence with census tract or zip-code level AER, parcel-level estimation provided a clearer view of the building infrastructure and reduced measurement errors in structural AER estimation due to spatial resolution. Moreover, with 16 years of data, we gained power even with further stratification by season and building characteristics that regulate AER and identified heterogeneities by season and housing factors.

Our results also suggest that future studies aiming to identify at-risk subpopulations exposed to ambient pollutants should consider exposure variability as a result of season and building or housing factors, especially when the residential conditions of the target population have a socioeconomic pattern.

Although this study added to the evidence of effect heterogeneity by ventilation and building factors, these factors vary by geographic region.^11,32^ Therefore, studies outside of MA may be comparable but should also consider the residential demographics inside different building characteristics as well as indoor sources of PM_2.5_.

## Conclusion

Parcel-level AER modifies the association between short-term ambient PM_2.5_ and mortality during warm seasons. Higher AER was associated with an increase in PM_2.5_-associated mortality. Including parcel-level AER may help us better quantify associations with ambient-sourced pollution by reducing measurement error, especially when it is differential. Future studies should also look to improve the AER models by applying a better spatial resolution and considering other influential factors that are not included in structural AERs.

## Conflicts of interest statement

The authors declare that they have no conflicts of interest with regard to the content of this report.

## Acknowledgments

We thank Dr. Anna Rosofsky for providing key information and data on her air exchange rate models. We thank Dr. Joel Schwartz’s group for sharing study data and providing computing environments.

## Supplementary Material
