# Supplementary material for: Short-term ambient PM2.5 exposure and cause-specific mortality in Massachusetts: Effect modification by structural air exchange rates
Source: Environ Epidemiol. 2025 Apr 16;9(3):e385. doi: 10.1097/EE9.0000000000000385 (PMC12005940; doi:10.1097/EE9.0000000000000385)
Supplement: Supplementary file 1 [file ee9-9-e385-s001.pdf]

Supplementary materials

Short-term Ambient $PM_{2.5}$ Exposure and Cause-specific Mortality in Massachusetts: Effect Modification by Structural Air Exchange Rates

Futu Chen[a], Jaime E. Hart[a,b], Jarvis T. Chen[c], Brent A. Coull[a,d], M. Patricia Fabian[e, f],Joel Schwartz[a,b], Gary Adamkiewicz[a]

[a] Department of Environmental Health, Harvard T.H. Chan School of Public Health, Boston, MA, USA
[b] Channing Division of Network Medicine, Department of Medicine, Brigham and Hospital and Harvard Medical School, Boston, MA, USA
[c] Department of Social and Behavioral Sciences, Harvard T. H. Chan School of Public Health, Boston, MA, USA
[d] Department of Biostatistics, Harvard T. H. Chan School of Public Health, Boston, MA, USA
[e] Department of Environmental Health, Boston University School of Public Health, Boston, MA, USA

Table of Contents

Figure S.1. Comparison of spatial units (2010 census tracts, census block groups, census blocks and parcels), South End neighborhood, Boston, MA

Supplemental methods for calculating non-residential air exchange rate (NR-AER)

Table S.1. Illustration of residential and non-residential real estate match and shelter types

Figure S.2. Flow chart of inclusion of deaths, 2000-2015, MA

Figure S.3. Comparison of residential AER and NR-AER by season

Table S.2. AER (1/hr) estimates comparing residential AER with NR-AER by season

Figure S.4.a Census tract crude mortality rate (total non-accidental all-cause mortality in the study/1000 population) by season, MA, 2010

Figure S.4.b Average parcel air exchange rate (AER, 1/hr) included in the study by season by census tract, MA, 2010

Figure S.4.c Crude mortality rate and average parcel air exchange rate (AER) included in the study by season by census tract, Suffolk County, MA, 2010

Table S.3. Odds ratios and 95% CI for the association between $PM_{2.5}$ and all-cause mortality on different exposure windows

Table S.4.a Odds ratios and 95% CI from generalized additive models with smooth term to test linearity of the main effect during warm season

Table S.4.b Odds ratios and 95% CI from generalized additive models with smooth term to test linearity of the main effect during cool season

Table S.5.a Odds ratios and 95% CI from generalized additive models with smooth term to test linearity of the effect modification by AER during warm season

Table S.5.b Odds ratios and 95% CI from generalized additive models with smooth term to test linearity of the effect modification by AER during cool season

Table S.6.a. Odds ratios and 95% for conditional logistic regression with AER*PM product term by cause-specific mortality during warm season

Table S.6.b. Odds ratios and 95% for conditional logistic regression with AER*PM product term by cause-specific mortality during cool season

Table S.7.a. Odds ratios and 95% for conditional logistic regression with AER*PM product term by cause-specific mortality during warm season, stratified by housing type

Table S.7.b. Odds ratios and 95% for conditional logistic regression with AER*PM product term by cause-specific mortality during cool season, stratified by housing type

Table S.7.c. Odds ratios and 95% for conditional logistic regression with AER*PM product term by cause-specific mortality during warm season, stratified by parcel residential type

Table S.7.d. Odds ratios and 95% for conditional logistic regression with AER*PM product term by cause-specific mortality during cool season, stratified by parcel residential type

Table S.8.a. Odds ratios and 95% for conditional logistic regression with AER*PM product term by cause-specific mortality during warm season, when the ambient temperature of the death day is less than 29 ^{o}C, by housing type

Table S.8.b. Odds ratios and 95% for conditional logistic regression with AER*PM product term by cause-specific mortality during warm season, when the ambient temperature of the death day is less than 29 ^{o}C, by parcel residential type

Table S.9.a. Odds ratios and 95% for conditional logistic regression with AER*PM product term by cause-specific mortality during warm season, excluding both in-patient and outpatient in-hospital death, by housing type

Table S.9.b. Odds ratios and 95% for conditional logistic regression with AER*PM product term by cause-specific mortality during cool season, excluding both in-patient and outpatient in-hospital death, by housing type

Table S.9.c. Odds ratios and 95% for conditional logistic regression with AER*PM product term by cause-specific mortality during warm season excluding both in-patient and outpatient in-hospital death, by parcel residential type

Table S.9.d. Odds ratios and 95% for conditional logistic regression with AER*PM product term by cause-specific mortality during cool season excluding both in-patient and outpatient in-hospital death, by parcel residential type

Figure S.5. Estimated percentage change in cause-specific mortality associated with a 10μg/m^3 increase in $PM_{2.5}$ at 15% and 75% AER, stratified by season, parcel housing and residential type, excluding both in-patient and outpatient in-hospital death, 2000-2015, Massachusetts, USA.

Figure S.1. Comparison of spatial units (2010 census tracts, census block groups, census blocks and parcels), South End neighborhood, Boston, MA

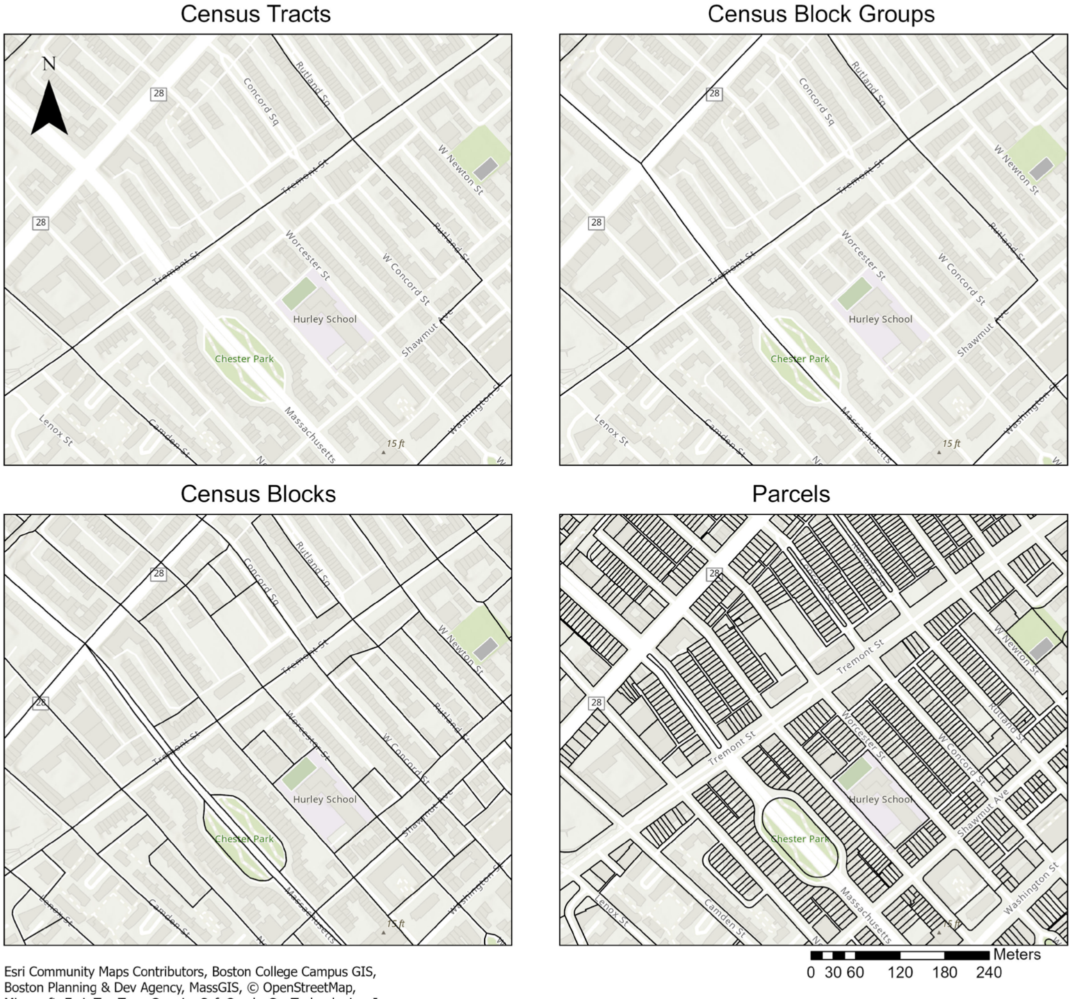

Esri Community Maps Contributors, Boston College Campus GIS, Boston Planning & Dev Agency, MassGIS, © OpenStreetMap, Microsoft, Esri, TomTom, Garmin, SafeGraph, GeoTechnologies, Inc, METI/NASA, USGS, EPA, NPS, US Census Bureau, USDA, USFWS, Sources: Esri, Airbus DS, USGS, NGA, NASA, CGIAR, N Robinson, NCEAS, NLS, OS, NMA, Geodatastyrelsen, Rijkswaterstaat, GSA, Geoland, FEMA, Intermap and the GIS user community

Figure S.2. Flow chart of inclusion of deaths, 2000-2015, MA

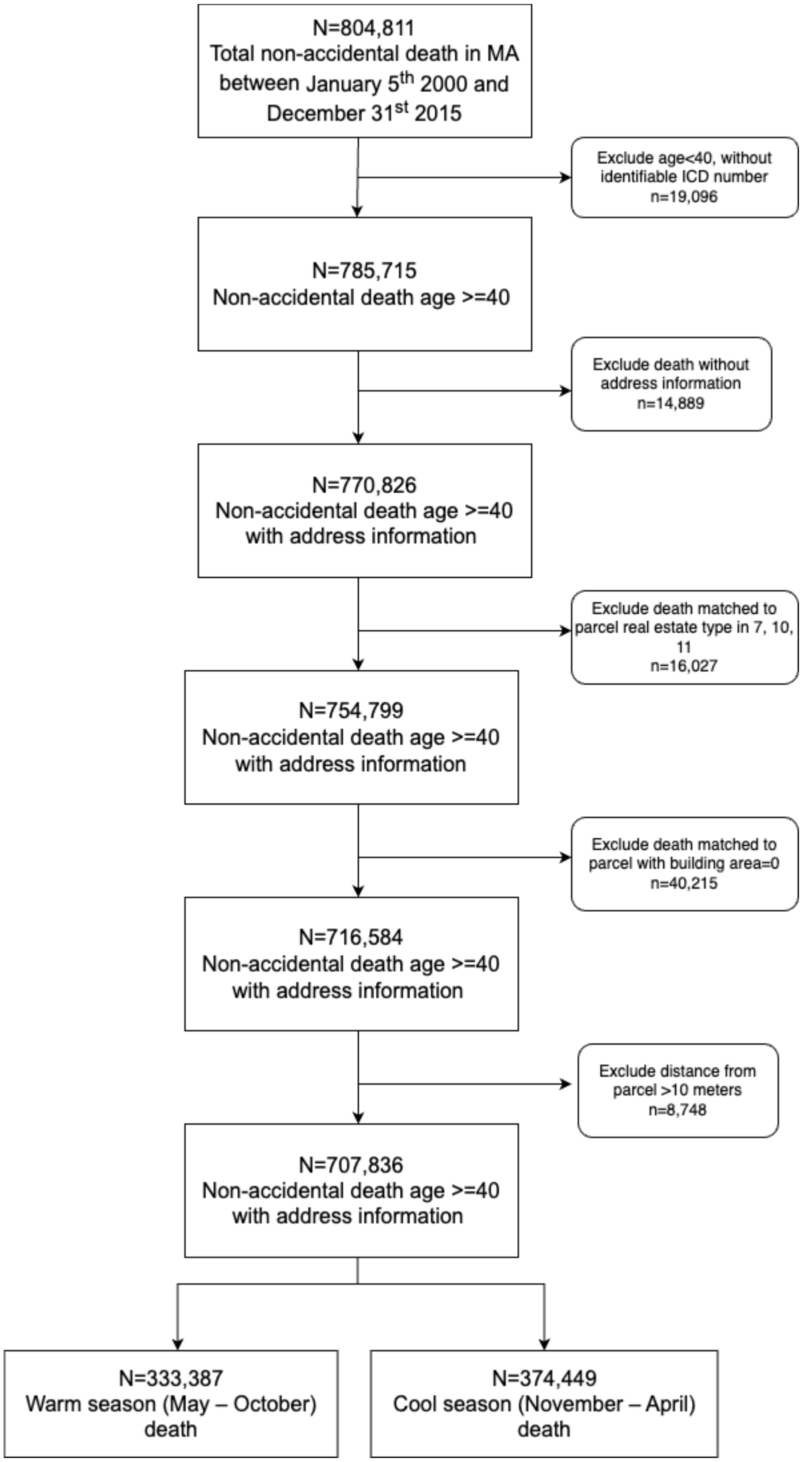

Supplemental material for calculating non-residential air exchange rate (NR-AER)

In general, we followed Rosofsky et al. (2019) for the same method and same data source. We documented key discrepancies in our approaches here. For information that do not need to be repeated in this supplemental, please refer to supporting information under Rosofsky et al. (2019).

Determination of parcels:
For our analysis, we retrieved non-residential parcel data from MAPC (2016). We first excluded parcels that did not have a real estate type (5.5% of total non-residential parcels). These parcels were utilities, driveways, and open areas such as lakes and gardens.
We then merged residential parcel Rosofsky et al. (2019) used (where parcels had a real estate type of 1-5) and reduced non-residential parcels (where parcels were assigned a different real estate type by MAPC). We performed a spatial join (closest join) from death to the merged parcel shapefile. After joining, 48,511 (9.94%) non-residential parcels were joined with at least one mortality. We then only modeled NR-AER for those parcels that contained a death from 2000-2015. We also excluded those parcels that contained a building area of $0m^2$ even after imputation.

Steps of calculation:
The critical difference between residential AER and non-residential AER calculations were the definition of shelter class, which serves as "a surrogate of wind shielding from surrounding obstructions". It was determined by the surrounding land use as well as housing structure (single-family, duplex/triplex, multifamily buildings). Rosofsky et al. (2019) assigned all parcels categorized as multi-family a shelter class of "5" to accommodate the fact that apartments have fewer outer walls and hence less contact with outdoor wind and temperature.

To approximate residential parcels with non-residential parcel real estate types, we use the following approach:

1) Exclude real estate type 7 (agriculture and outdoor recreational activities), 10 (educational uses such as universities), and 11 (industrial properties, warehouse and utilities), because these were less likely to be a residential address and also have extreme values such as building area.
2) If the owner of the parcel contained key word "housing authority", "City of", and "Charity", we assumed them to be either nursing homes or public housings, and were multifamily buildings. We assigned them as multifamily- equivalent parcels.
3) For the rest of the parcels included, if parcel real estate type belonged to 0 (unknown), 6 (mixed use: more than half commercial use), 8 (commercial, retail, entertainment and offices with floor area ratio, FAR < 0.75), 9 (commercial, retail, entertainment and offices with FAR of 0.75 or more), and 12 (tax exempt properties), we assigned their parcel residential type by the estimated units. If estimated unites <=1, we assigned them as single family- equivalent parcels. If estimated units were from 1 to 2 (including 2 units), we assigned them as duplex/triplex-equivalent parcels. If estimated units was >2, we assigned them as multifamily-equivalent parcels.

Table S.1. Illustration of residential and non-residential real estate match and shelter types

Residential type	Non-residential type	Shelter Class
1: Single family properties	0, 6, 8, 9, 12 with estimated unites <=1	Depend on land use (1-5)
2: Duplex/triplex	0, 6, 8, 9, 12 with estimated unites $\in (1,2]$	Depend on land use (1-5)
3: Small apartments	0, 6, 8, 9, 12 with estimated unites >2; Or identified as public housings, city properties, nursing homes.	5
4: Large apartments	0, 6, 8, 9, 12 with estimated unites >2; Or identified as public housings, city properties, nursing homes.	5
5: Multi-use residential	0, 6, 8, 9, 12 with estimated unites >2; Or identified as public housings, city properties, nursing homes.	5

Figure S.2. Comparison of residential AER and NR-AER by season, MA parcels

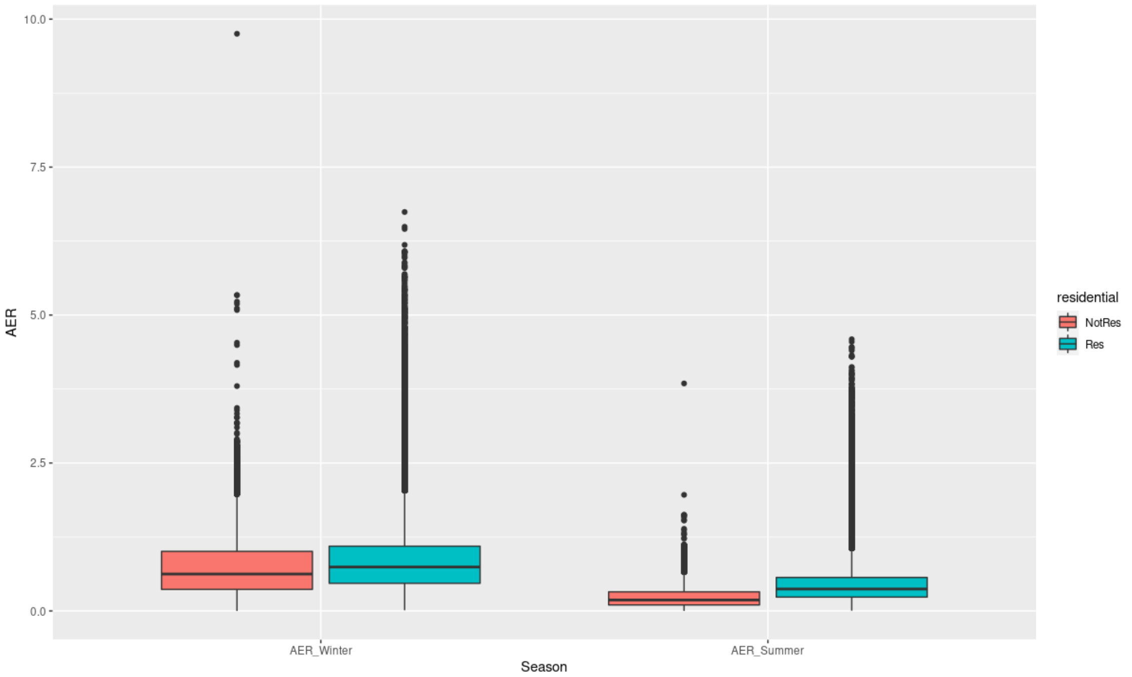

Figure S.3.a Census tract crude mortality rate (total non-accidental all-cause mortality in the study/1000 population) by season, MA, 2010

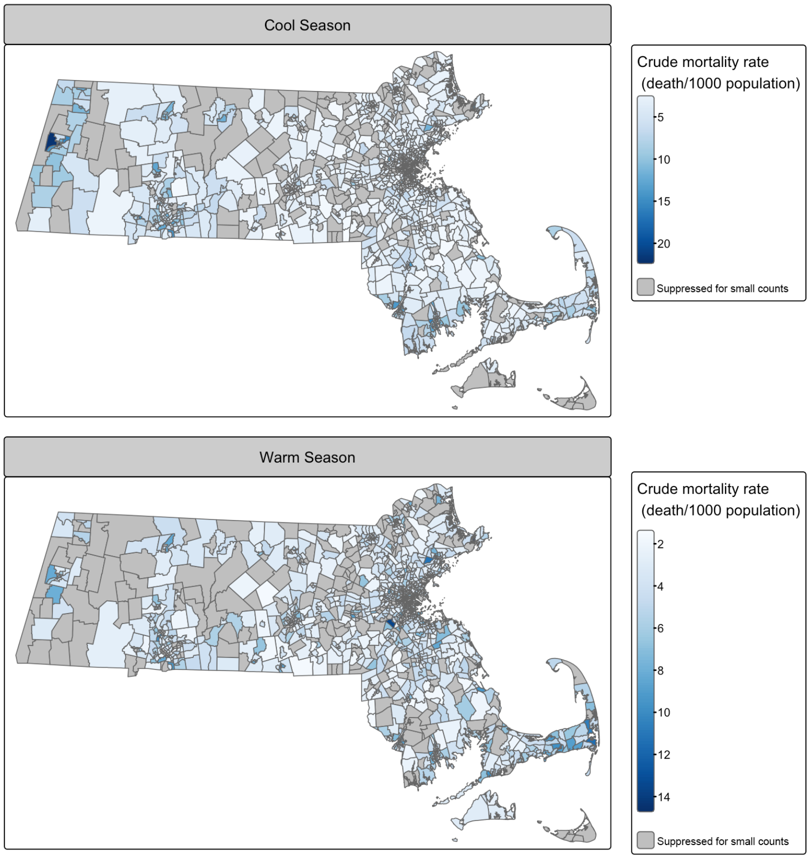

Note: Crude mortality rate=sum(non-accidental all-cause mortality cases included in 2010 in the study)/total population in 2010 by census tract. Baseline population data from 2010 decennial census. Small cells (total death <=10) were suppressed.

Figure S.3.b Average parcel air exchange rate (AER, 1/hr) included in the study by season by census tract, MA, 2010

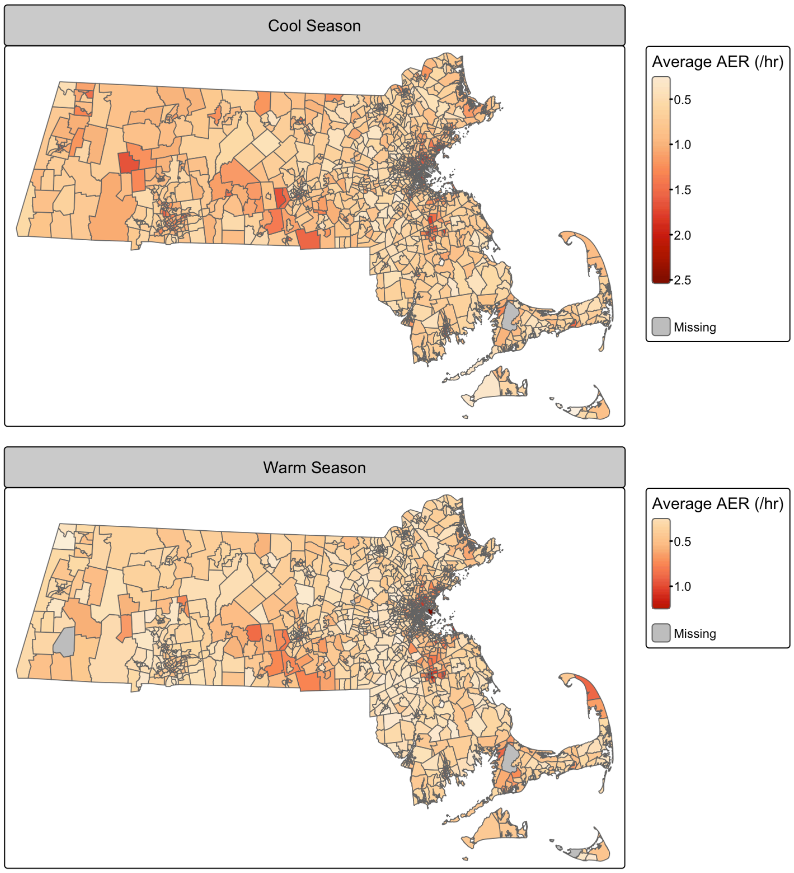

Figure S.3.c Crude mortality rate and average parcel air exchange rate (AER) included in the study by season by census tract, Suffolk County, MA, 2010

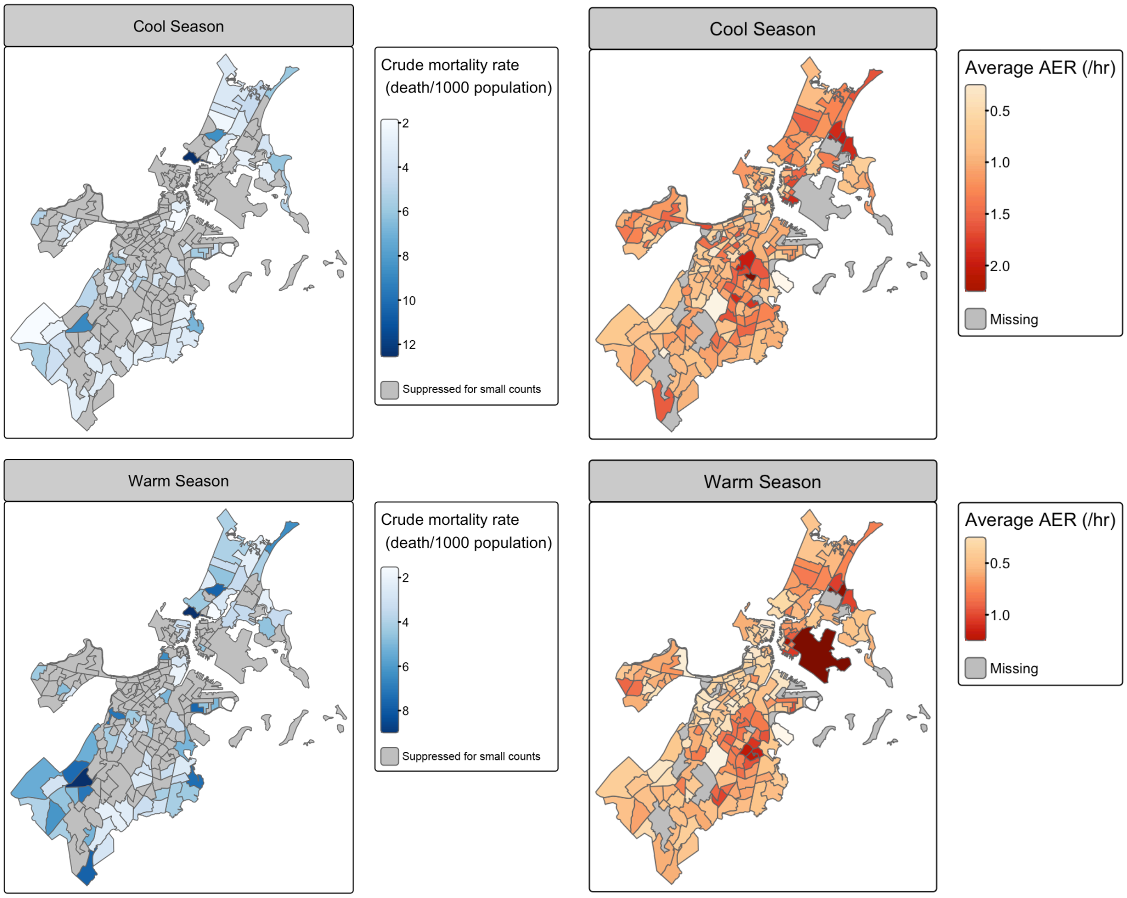

Note: Crude mortality rate=sum(non-accidental all-cause mortality cases included in 2010 in the study)/total population in 2010 by census tract. Baseline population data from 2010 decennial census. Small cells (total death <=10) were suppressed.

Table S.2. AER (1/hr) estimates comparing residential AER with NR-AER by season, MA parcels

**Residential AERs (Winter)**					
Min	1st Qu.	Median	Mean	3rd Qu.	Max
0.0117	0.4671	0.7422	0.8463	1.0947	6.7415
**Residential AERs (Summer)**					
Min	1st Qu.	Median	Mean	3rd Qu.	Max
0.0043	0.234	0.3711	0.4468	0.5657	4.5912

**Non-Residential AERs that joined to a death (Winter)**					
Min	1st Qu.	Median	Mean	3rd Qu.	Max
<0.00001	0.197	0.425	0.552	0.756	9.753
**Non-Residential AERs that joined to a death (Summer)**					
Min	1st Qu.	Median	Mean	3rd Qu.	Max
<0.00001	0.0544	0.1349	0.1805	0.2432	3.844

Table S.3. Odds ratios and 95% CI for the association between $PM_{2.5}$ and all-cause mortality on different exposure windows.

**Exposure window**	**$PM_{2.5}$ (10µg/m^3 increase) N=7,797,868**	
	Odds Ratio	95% CI
Day 0	1.012	(1.007,1.017)
Day 1	1.017	(1.012,1.022)
Day 2	1.008	(1.003,1.012)
Day 3	1.004	(0.999,1.009)
Day 4	1.001	(0.996,1.005)
Day 0-1	1.020	(1.014,1.026)
Day 0-2	1.022	(1.015,1.028)
Day 0-3	1.022	(1.014,1.029)
Day 0-4	1.019	(1.011,1.028)
Day 1-2	1.016	(1.01,1.022)
Day 1-3	1.015	(1.008,1.022)
Day 1-4	1.012	(1.005,1.02)
Day 2-3	1.007	(1.001,1.013)
Day 2-4	1.004	(0.998,1.011)
Day 3-4	1.002	(0.996,1.008)

Note: models additionally controlled for day of the week, daily average temperature and relative humidity using the same exposure window as $PM_{2.5}$

Table S.4.a Odds ratios and 95% CI from generalized additive models with smooth term to test linearity of the main effect during warm season

	All-Cause Mortality N=333,387		CVD Mortality N=110,256		Respiratory Mortality N=34,239	
Predictors	*Odds Ratio (95% CI)*	*P-Value*	*Odds Ratio (95% CI)*	*P-Value*	*Odds Ratio (95% CI)*	*P-Value*
$PM_{2.5}$ (10μg/m^3 increase)	1.01 (0.97 – 1.04)	0.701	1.02 (1.00 – 1.04)	**0.021**	1.02 (0.99 – 1.05)	0.211
Temperature (10°C increase)	1 (0.99 – 1.02)	0.597	1 (0.97 – 1.02)	0.941	0.98 (0.94 – 1.02)	0.332
RH (10% increase)	1 (0.99 – 1.00)	0.129	0.99 (0.98 – 1.00)	0.14	0.99 (0.98 – 1.01)	0.355
s(PM, m=c(2, 0))		0.056		0.841		0.796

Table S.4.b Odds ratios and 95% CI from generalized additive models with smooth term to test linearity of the main effect during cool season

	All-Cause Mortality N=374,449		CVD Mortality N=126,064		Respiratory Mortality N=44,290	
Predictors	*Odds Ratio (95% CI)*	*P-Value*	*Odds Ratio (95% CI)*	*P-Value*	*Odds Ratio (95% CI)*	*P-Value*
$PM_{2.5}$ (10μg/m^3 increase)	1.02 (1.01 – 1.03)	**0.001**	1.01 (0.99 – 1.04)	0.341	1.01 (0.98 – 1.04)	0.469
Temperature (10°C increase)	1 (0.99 – 1.01)	0.764	0.99 (0.97 – 1.00)	0.107	1.02 (0.99 – 1.05)	0.152
RH (10% increase)	1.01 (1.00 – 1.01)	**<0.001**	1.01 (1.01 – 1.02)	**<0.001**	1.01 (0.99 – 1.02)	0.386
s(PM, m=c(2, 0))		0.792		0.348		0.693

Note: s(PM, m=c(2,0)) indicated a smooth term of PM without penalty of the null space but only penalty on the 2nd derivative of the smooth that measured wiggliness
Abbreviation: CI=confidence interval, RH=relative humidity

Table S.5.a Odds ratios and 95% CI from generalized additive models with smooth term to test linearity of the effect modification by AER during warm season

	All-Cause Mortality		CVD Mortality N=110,256		Respiratory Mortality N=34,239	
Predictors	*Odds Ratio (95% CI)*	*P-Value*	*Odds Ratio (95% CI)*	*P-Value*	*Odds Ratio (95% CI)*	*P-Value*
$PM_{2.5}$ (10μg/m^3 increase)			1.01 (0.66 – 1.55)	0.954	0.99 (0.87 – 1.13)	0.936
Temperature (10°C increase)			1 (0.97 – 1.02)	0.949	0.98 (0.94 – 1.02)	0.334
RH (10% increase)			0.99 (0.98 – 1.00)	0.14	0.99 (0.98 – 1.01)	0.355
s(AER by PM)		na		0.245		0.427

Table S.5.b Odds ratios and 95% CI from generalized additive models with smooth term to test linearity of the effect modification by AER during cool season

	All-Cause Mortality		CVD Mortality N=126,064		Respiratory Mortality N=44,239	
Predictors	*Odds Ratio (95% CI)*	*P-Value*	*Odds Ratio (95% CI)*	*P-Value*	*Odds Ratio (95% CI)*	*P-Value*
$PM_{2.5}$ (10μg/m^3 increase)			1.02 (0.87 – 1.20)	0.773	1.1 (0.71 – 1.70)	0.663
Temperature (10°C increase)			0.99 (0.97 – 1.00)	0.1	1.02 (0.99 – 1.05)	0.151
RH (10% increase)			1.01 (1.01 – 1.02)	**<0.001**	1.01 (0.99 – 1.02)	0.39
s(AER by PM)		na		0.822		0.304

Note: s(AER by PM) indicated a smooth term of AER by linear PM main effect; due to computational time, all-cause models are NA.
Abbreviation: CI=confidence interval, RH=relative humidity

Table S.6.a. Odds ratios and 95% for conditional logistic regression with AER*PM product term by cause-specific mortality during warm season

	**All-Cause Mortality N=3,399,415**			**CVD Mortality N=1,124,236**			**Respiratory Mortality N=3349,044**		
Predictors	*Odds Ratio*	*Conf. Int (95%)*	*P-Value*	*Odds Ratio*	*Conf. Int (95%)*	*P-Value*	*Odds Ratio*	*Conf. Int (95%)*	*P-Value*
$PM_{2.5}$ (10μg/m^3 increase)	1.01	1.00 – 1.03	**0.046**	1.02	0.99 – 1.04	0.133	1.00	0.96 – 1.04	0.970
Temperature (10°C increase)	1.01	0.99 – 1.02	0.220	1.00	0.97 – 1.02	0.943	0.98	0.94 – 1.02	0.335
RH (10% increase)	1.00	0.99 – 1.00	0.093	0.99	0.98 – 1.00	0.140	0.99	0.98 – 1.01	0.355
$PM_{2.5}$ *AER	1.01	0.99 – 1.04	0.249	1.01	0.97 – 1.05	0.653	1.05	0.98 – 1.12	0.199

Table S.6-b. Odds ratios and 95% for conditional logistic regression with AER*PM product term by cause-specific mortality during cool season

	**All-Cause Mortality N=3,761,308**			**CVD Mortality N=1,265,929**			**Respiratory Mortality N=444,574**		
Predictors	*Odds Ratio*	*Conf. Int (95%)*	*P-Value*	*Odds Ratio*	*Conf. Int (95%)*	*P-Value*	*Odds Ratio*	*Conf. Int (95%)*	*P-Value*
$PM_{2.5}$ ($10 \mu g/m^3$ increase)	1.03	1.01 – 1.04	**0.001**	1.02	0.99 – 1.05	0.121	1.04	0.99 – 1.09	0.101
Temperature (10°C increase)	1.00	0.99 – 1.01	0.766	0.99	0.97 – 1.00	0.100	1.02	0.99 – 1.05	0.152
RH (10% increase)	1.01	1.00 – 1.01	**<0.001**	1.01	1.01 – 1.02	**<0.001**	1.01	0.99 – 1.02	0.390
$PM_{2.5}$ *AER	0.99	0.98 – 1.01	0.421	0.99	0.97 – 1.02	0.529	0.97	0.92 – 1.01	0.146

Table S.7.a. Odds ratios and 95% for conditional logistic regression with AER*PM product term by cause-specific mortality during warm season, stratified by housing type (SF=Single family parcels; MF=Multifamily parcels)

	**All-Cause Mortality (SF, N=2,075,209)**			**CVD Mortality (SF, N=686,003)**			**Respiratory Mortality (SF, N=2,090,51)**			**All-Cause Mortality (MF, N=1,324,206)**			**CVD Mortality (MF, N=438,233)**			**Respiratory Mortality (MF, N=139,993)**		
Predictors	*Odds Ratio*	*Conf. Int (95%)*	*P-Value*	*Odds Ratio*	*Conf. Int (95%)*	*P-Value*	*Odds Ratio*	*Conf. Int (95%)*	*P-Value*	*Odds Ratio*	*Conf. Int (95%)*	*P-Value*	*Odds Ratio*	*Conf. Int (95%)*	*P-Value*	*Odds Ratio*	*Conf. Int (95%)*	*P-Value*
$PM_{2.5}$ (10μg/m^3 increase)	1.03	1.01 – 1.04	**0.004**	1.02	0.99 – 1.05	0.110	1.04	0.99 – 1.10	0.132	1.00	0.98 – 1.02	0.835	1.01	0.97 – 1.04	0.752	0.96	0.90 – 1.02	0.199
Temperature (10ºC increase)	1.00	0.98 – 1.02	0.983	0.99	0.96 – 1.02	0.484	0.99	0.93 – 1.05	0.655	1.02	1.00 – 1.05	0.054	1.02	0.98 – 1.06	0.443	0.97	0.90 – 1.04	0.361
RH (10% increase)	1.00	0.99 – 1.00	0.534	0.99	0.98 – 1.00	0.230	0.99	0.97 – 1.01	0.313	0.99	0.98 – 1.00	0.060	0.99	0.98 – 1.01	0.388	1.00	0.97 – 1.02	0.793
$PM_{2.5}$*AER	0.99	0.96 – 1.03	0.670	1.01	0.95 – 1.07	0.831	0.94	0.84 – 1.04	0.240	1.03	1.00 – 1.06	**0.044**	1.01	0.96 – 1.07	0.633	1.15	1.05 – 1.27	**0.004**

Table S.7.b. Odds ratios and 95% for conditional logistic regression with AER*PM product term by cause-specific mortality during cool season, stratified by housing type (SF=Single family parcels; MF=Multifamily parcels)

	**All-Cause Mortality (SF, N=2,300,814)**			**CVD Mortality (SF, N=770,962)**			**Respiratory Mortality (SF, N=268,065)**			**All-Cause Mortality (MF, N=1,460,494)**			**CVD Mortality (MF, N=494,967)**			**Respiratory Mortality (MF, N=176,509)**		
Predictors	*Odds Ratio*	*Conf. Int (95%)*	*P-Value*	*Odds Ratio*	*Conf. Int (95%)*	*P-Value*	*Odds Ratio*	*Conf. Int (95%)*	*P-Value*	*Odds Ratio*	*Conf. Int (95%)*	*P-Value*	*Odds Ratio*	*Conf. Int (95%)*	*P-Value*	*Odds Ratio*	*Conf. Int (95%)*	*P-Value*
$PM_{2.5}$ (10µg/m^3 increase)	1.03	1.01 – 1.05	**0.015**	1.00	0.97 – 1.04	0.934	1.04	0.98 – 1.11	0.170	1.02	1.00 – 1.05	0.065	1.04	1.00 – 1.09	0.058	1.01	0.93 – 1.08	0.866
Temperature (10ºC increase)	1.00	0.99 – 1.01	0.800	1.00	0.98 – 1.02	0.639	1.03	0.99 – 1.06	0.135	1.00	0.99 – 1.02	0.881	0.97	0.95 – 1.00	**0.042**	1.01	0.97 – 1.05	0.666
RH (10% increase)	1.01	1.00 – 1.01	**0.001**	1.01	1.00 – 1.02	**0.019**	1.00	0.99 – 1.02	0.573	1.01	1.00 – 1.01	0.113	1.02	1.01 – 1.03	**0.002**	1.01	0.99 – 1.02	0.511
$PM_{2.5}$ *AER	0.99	0.97 – 1.02	0.657	1.02	0.98 – 1.06	0.420	0.98	0.92 – 1.06	0.651	0.99	0.97 – 1.02	0.594	0.97	0.94 – 1.01	0.134	0.97	0.91 – 1.04	0.398

Table S.7.c. Odds ratios and 95% for conditional logistic regression with AER*PM product term by cause-specific mortality during warm season, stratified by parcel residential type (Res=Residential parcels; NotRes=Non-residential parcels)

	**All-Cause Mortality (Res, N=2,468,570)**			**CVD Mortality (Res, N=806,754)**			**Respiratory Mortality (Res, N=241,153)**			**All-Cause Mortality (NotRes, N=930,845)**			**CVD Mortality (NotRes, N=317,482)**			**Respiratory Mortality (NotRes, N=107, 891)**		
Predictors	*Odds Ratio*	*Conf. Int (95%)*	*P-Value*	*Odds Ratio*	*Conf. Int (95%)*	*P-Value*	*Odds Ratio*	*Conf. Int (95%)*	*P-Value*	*Odds Ratio*	*Conf. Int (95%)*	*P-Value*	*Odds Ratio*	*Conf. Int (95%)*	*P-Value*	*Odds Ratio*	*Conf. Int (95%)*	*P-Value*
$PM_{2.5}$ (10μg/m^3 increase)	1.01	0.99 – 1.03	0.330	1.01	0.99 – 1.04	0.344	0.97	0.92 – 1.03	0.284	1.02	0.99 – 1.04	0.191	1.01	0.98 – 1.05	0.505	1.05	0.98 – 1.12	0.162
Temperature (10ºC increase)	1.01	0.99 – 1.02	0.467	1.00	0.97 – 1.03	0.918	0.98	0.93 – 1.03	0.447	1.02	0.99 – 1.04	0.255	1.00	0.96 – 1.05	0.991	0.98	0.90 – 1.06	0.564
RH (10% increase)	1.00	0.99 – 1.00	0.431	1.00	0.98 – 1.01	0.391	0.99	0.97 – 1.01	0.265	0.99	0.98 – 1.00	0.057	0.99	0.97 – 1.00	0.161	1.00	0.97 – 1.03	0.994
$PM_{2.5}$ *AER	1.02	0.99 – 1.05	0.152	1.01	0.97 – 1.06	0.673	1.10	1.01 – 1.19	**0.035**	1.03	0.95 – 1.12	0.445	1.07	0.94 – 1.22	0.336	0.90	0.71 – 1.14	0.361

Table S.7.d. Odds ratios and 95% for conditional logistic regression with AER*PM product term by cause-specific mortality during cool season, stratified by parcel residential type (Res=Residential parcels; NotRes=Non-residential parcels)

	**All-Cause Mortality (Res, N=2,714,181)**			**CVD Mortality (Res, N=907,223)**			**Respiratory Mortality (Res, N=308,586)**			**All-Cause Mortality (NotRes, N=1,047,127)**			**CVD Mortality (NotRes, N=358,706)**			**Respiratory Mortality (NotRes), N=135,988**		
Predictors	*Odds Ratio*	*Conf. Int (95%)*	*P-Value*	*Odds Ratio*	*Conf. Int (95%)*	*P-Value*	*Odds Ratio*	*Conf. Int (95%)*	*P-Value*	*Odds Ratio*	*Conf. Int (95%)*	*P-Value*	*Odds Ratio*	*Conf. Int (95%)*	*P-Value*	*Odds Ratio*	*Conf. Int (95%)*	*P-Value*
$PM_{2.5}$ (10µg/m^3 increase)	1.02	0.99 – 1.04	0.144	1.02	0.98 – 1.06	0.348	1.04	0.98 – 1.11	0.180	1.04	1.01 – 1.07	**0.002**	1.03	0.99 – 1.07	0.198	1.03	0.97 – 1.11	0.337
Temperature (10ºC increase)	1.00	0.99 – 1.01	0.502	0.99	0.97 – 1.01	0.340	1.02	0.99 – 1.05	0.258	1.00	0.98 – 1.01	0.623	0.98	0.95 – 1.01	0.118	1.02	0.97 – 1.07	0.378
RH (10% increase)	1.01	1.00 – 1.01	**0.030**	1.01	1.00 – 1.02	**0.018**	1.01	0.99 – 1.02	0.250	1.01	1.01 – 1.02	**0.001**	1.02	1.01 – 1.04	**0.001**	1.00	0.98 – 1.02	0.862
$PM_{2.5}$ *AER	1.00	0.98 – 1.02	0.985	0.99	0.96 – 1.03	0.743	0.96	0.91 – 1.02	0.201	0.99	0.96 – 1.02	0.552	0.99	0.93 – 1.04	0.610	0.97	0.88 – 1.07	0.510

Table S.8.a. Odds ratios and 95% for conditional logistic regression with AER*PM product term by cause-specific mortality during warm season, when the ambient temperature of the death day is less than 29 °C, by housing type (SF=Single family parcels; MF=Multifamily parcels)

	**All-Cause Mortality (SF, N=2,071,395)**			**CVD Mortality (SF, N=684,790)**			**Respiratory Mortality (SF, N=208,686)**			**All-Cause Mortality (MF, N=1,321,008)**			**CVD Mortality (MF, N=437,212)**			**Respiratory Mortality (MF, N=139,673)**		
Predictors	*Odds Ratio*	*Conf. Int (95%)*	*P-Value*	*Odds Ratio*	*Conf. Int (95%)*	*P-Value*	*Odds Ratio*	*Conf. Int (95%)*	*P-Value*	*Odds Ratio*	*Conf. Int (95%)*	*P-Value*	*Odds Ratio*	*Conf. Int (95%)*	*P-Value*	*Odds Ratio*	*Conf. Int (95%)*	*P-Value*
$PM_{2.5}$ (10μg/m^3 increase)	1.03	1.01 – 1.04	**0.003**	1.02	1.00 – 1.05	0.103	1.04	0.99 – 1.10	0.122	1.00	0.98 – 1.02	0.955	1.01	0.97 – 1.04	0.687	0.96	0.91 – 1.03	0.252
Temperature (10°C increase)	1.00	0.98 – 1.02	0.979	0.99	0.96 – 1.02	0.505	0.98	0.93 – 1.04	0.583	1.02	1.00 – 1.04	0.073	1.01	0.97 – 1.05	0.503	0.96	0.90 – 1.03	0.307
RH (10% increase)	1.00	0.99 – 1.00	0.512	0.99	0.98 – 1.00	0.203	0.99	0.97 – 1.01	0.328	0.99	0.98 – 1.00	**0.068**	0.99	0.98 – 1.01	0.376	1.00	0.97 – 1.02	0.851
$PM_{2.5}$ *AER	0.99	0.96 – 1.03	0.626	1	0.95 – 1.06	0.899	0.94	0.84 – 1.05	0.251	1.03	1.00 – 1.06	0.067	1.01	0.96 – 1.07	0.702	1.15	1.05 – 1.27	**0.004**

Table S.8.b. Odds ratios and 95% for conditional logistic regression with AER*PM product term by cause-specific mortality during warm season, when the ambient temperature of the death day is less than 29 °C, by parcel residential type (Res=Residential parcels; NotRes=Non-residential parcels)

	**All-Cause Mortality (Res, N=2,463,516)**			**CVD Mortality (Res, N=805,165)**			**Respiratory Mortality (Res, N=240,674)**			**All-Cause Mortality (NotRes, N=928,997)**			**CVD Mortality (NotRes, N=316,837)**			**Respiratory Mortality (NotRes, N=107,685)**		
Predictors	*Odds Ratio*	*Conf. Int (95%)*	*P-Value*	*Odds Ratio*	*Conf. Int (95%)*	*P-Value*	*Odds Ratio*	*Conf. Int (95%)*	*P-Value*	*Odds Ratio*	*Conf. Int (95%)*	*P-Value*	*Odds Ratio*	*Conf. Int (95%)*	*P-Value*	*Odds Ratio*	*Conf. Int (95%)*	*P-Value*
$PM_{2.5}$ (10μg/m^3 increase)	1.01	0.99 – 1.03	0.222	1.02	0.99 – 1.05	0.269	0.97	0.92 – 1.03	0.287	1.02	0.99 – 1.04	0.189	1.01	0.98 – 1.05	0.497	1.05	0.99 – 1.12	0.13
Temperature (10°C increase)	1.01	0.99 – 1.02	0.533	1.00	0.97 – 1.03	0.867	0.98	0.93 – 1.03	0.399	1.01	0.99 – 1.04	0.287	1.00	0.96 – 1.05	0.963	0.97	0.90 – 1.05	0.478
RH (10% increase)	1.00	0.99 – 1.00	0.418	0.99	0.98 – 1.01	0.354	0.99	0.97 – 1.01	0.289	0.99	0.98 – 1.00	0.064	0.99	0.97 – 1.00	0.155	1.00	0.97 – 1.03	0.971
$PM_{2.5}$ *AER	1.02	0.99 – 1.04	0.236	1.01	0.96 – 1.05	0.792	1.10	1.01 – 1.20	**0.029**	1.03	0.95 – 1.11	0.486	1.06	0.92 – 1.21	0.428	0.90	0.71 – 1.15	0.396

Table S.9.a. Odds ratios and 95% for conditional logistic regression with AER*PM product term by cause-specific mortality during warm season, excluding both in-patient and outpatient in-hospital death, by housing type (SF=Single family parcels; MF=Multifamily parcels)

	**All-Cause Mortality (SF, N=1,274,567)**			**CVD Mortality (SF, N=380,785)**			**Respiratory Mortality (SF, N=104,398)**		
Predictors	*Odds Ratio*	*Conf. Int (95%)*	*P-Value*	*Odds Ratio*	*Conf. Int (95%)*	*P-Value*	*Odds Ratio*	*Conf. Int (95%)*	*P-Value*
$PM_{2.5}$ (10μg/m^3 increase)	1.03	1.01 – 1.05	**0.007**	1.05	1.01 – 1.09	**0.011**	1.03	0.95 – 1.11	0.462
Temperature (10ºC increase)	1.02	1.00 – 1.05	0.055	1.02	0.97 – 1.06	0.440	1.02	0.94 – 1.10	0.682
RH (10% increase)	1.00	0.99 – 1.01	0.634	0.99	0.98 – 1.01	0.271	0.99	0.97 – 1.03	0.744
$PM_{2.5}$*AER	1.00	0.96 – 1.05	0.972	0.98	0.91 – 1.06	0.578	0.97	0.83 – 1.14	0.735

	**All-Cause Mortality (MF, N=769,384)**			**CVD Mortality (MF, N=240,918)**			**Respiratory Mortality (MF, N=66,487)**		
Predictors	*Odds Ratio*	*Conf. Int (95%)*	*P-Value*	*Odds Ratio*	*Conf. Int (95%)*	*P-Value*	*Odds Ratio*	*Conf. Int (95%)*	*P-Value*
$PM_{2.5}$ (10μg/m^3 increase)	0.99	0.97 – 1.02	0.648	0.99	0.94 – 1.03	0.603	0.94	0.86 – 1.02	0.149
Temperature (10ºC increase)	1.05	1.02 – 1.08	**0.001**	1.04	0.99 – 1.10	0.119	0.99	0.90 – 1.10	0.897
RH (10% increase)	0.99	0.98 – 1.00	0.116	0.99	0.97 – 1.01	0.377	1.00	0.96 – 1.04	0.936
$PM_{2.5}$*AER	1.05	1.00 – 1.09	**0.035**	1.07	1.00 – 1.15	0.063	1.27	1.10 – 1.46	**0.001**

Table S.9.b. Odds ratios and 95% for conditional logistic regression with AER*PM product term by cause-specific mortality during warm season, excluding both in-patient and outpatient in-hospital death, by housing type (SF=Single family parcels; MF=Multifamily parcels)

	**All-Cause Mortality (SF, N=1,386,318)**			**CVD Mortality (SF, N=425,153)**			**Respiratory Mortality (SF, N=130,041)**			**All-Cause Mortality (MF, N=834,561)**			**CVD Mortality (MF, N=267,501)**			**Respiratory Mortality (MF, N=79,919)**		
Predictors	*Odds Ratio*	*Conf. Int (95%)*	*P-Value*	*Odds Ratio*	*Conf. Int (95%)*	*P-Value*	*Odds Ratio*	*Conf. Int (95%)*	*P-Value*	*Odds Ratio*	*Conf. Int (95%)*	*P-Value*	*Odds Ratio*	*Conf. Int (95%)*	*P-Value*	*Odds Ratio*	*Conf. Int (95%)*	*P-Value*
$PM_{2.5}$ (10μg/m^3 increase)	1.03	1.00 – 1.06	0.054	1.03	0.98 – 1.08	0.314	1.08	0.98 – 1.19	0.117	1.04	1.01 – 1.07	**0.012**	1.02	0.97 – 1.07	0.483	1.12	1.02 – 1.23	**0.013**
Temperature (10°C increase)	1.02	1.00 – 1.03	**0.021**	1.01	0.99 – 1.04	0.256	1.02	0.97 – 1.07	0.424	1.00	0.98 – 1.02	0.770	1.00	0.96 – 1.04	0.977	1.01	0.95 – 1.08	0.785
RH (10% increase)	1.01	1.00 – 1.01	0.061	1.01	0.99 – 1.02	0.310	1.02	1.00 – 1.04	0.133	1.02	1.01 – 1.03	**0.001**	1.03	1.01 – 1.05	**0.001**	1.01	0.98 – 1.04	0.546
$PM_{2.5}$*AER	0.99	0.97 – 1.02	0.589	1.00	0.95 – 1.04	0.833	0.96	0.88 – 1.05	0.342	1.00	0.96 – 1.04	0.975	1.00	0.93 – 1.07	0.966	0.90	0.79 – 1.03	0.133

Table S.9.c. Odds ratios and 95% for conditional logistic regression with AER*PM product term by cause-specific mortality during warm season excluding both in-patient and outpatient in-hospital death, by parcel residential type (Res=Residential parcels; NotRes=Non-residential parcels)

	**All-Cause Mortality (Res, N=1,440,380)**			**CVD Mortality (Res, N=422,129)**			**Respiratory Mortality (Res, N=111,716)**			**All-Cause Mortality (NotRes, N=603,571)**			**CVD Mortality (NotRes, N=199,574)**			**Respiratory Mortality (NotRes, N=59,169)**		
Predictors	*Odds Ratio*	*Conf. Int (95%)*	*P-Value*	*Odds Ratio*	*Conf. Int (95%)*	*P-Value*	*Odds Ratio*	*Conf. Int (95%)*	*P-Value*	*Odds Ratio*	*Conf. Int (95%)*	*P-Value*	*Odds Ratio*	*Conf. Int (95%)*	*P-Value*	*Odds Ratio*	*Conf. Int (95%)*	*P-Value*
$PM_{2.5}$ (10μg/m^3 increase)	1.01	0.99 – 1.03	0.395	1.02	0.98 – 1.06	0.314	0.94	0.86 – 1.02	0.114	1.02	0.99 – 1.05	0.246	1.03	0.98 – 1.08	0.272	1.06	0.97 – 1.16	0.167
Temperature (10ºC increase)	1.04	1.01 – 1.06	**0.002**	1.04	0.99 – 1.08	0.087	1.04	0.97 – 1.13	0.280	1.03	0.99 – 1.06	0.104	1.01	0.95 – 1.07	0.809	0.94	0.84 – 1.04	0.234
RH (10% increase)	1.00	0.99 – 1.00	0.367	0.99	0.98 – 1.01	0.356	0.98	0.96 – 1.01	0.253	0.99	0.98 – 1.01	0.281	0.99	0.97 – 1.01	0.261	1.02	0.98 – 1.06	0.281
$PM_{2.5}$*AER	1.03	0.99 – 1.06	0.167	1.02	0.96 – 1.09	0.536	1.19	1.05 – 1.35	**0.008**	1.07	0.97 – 1.19	0.155	1.06	0.90 – 1.26	0.486	0.87	0.63 – 1.22	0.425

Table S.9.d. Odds ratios and 95% for conditional logistic regression with AER*PM product term by cause-specific mortality during cool season excluding both in-patient and outpatient in-hospital death, by parcel residential type (Res=Residential parcels; NotRes=Non-residential parcels)

	**All-Cause Mortality (Res, N=1,548,764)**			**CVD Mortality (Res, N=468,535)**			**Respiratory Mortality (Res, N=137,672)**			**All-Cause Mortality (NotRes, N=672,115)**			**CVD Mortality (NotRes, N=224,119)**			**Respiratory Mortality (NotRes, N=72,288)**		
Predictors	*Odds Ratio*	*Conf. Int (95%)*	*P-Value*	*Odds Ratio*	*Conf. Int (95%)*	*P-Value*	*Odds Ratio*	*Conf. Int (95%)*	*P-Value*	*Odds Ratio*	*Conf. Int (95%)*	*P-Value*	*Odds Ratio*	*Conf. Int (95%)*	*P-Value*	*Odds Ratio*	*Conf. Int (95%)*	*P-Value*
$PM_{2.5}$ (10μg/m^3 increase)	1.03	1.00 – 1.06	0.054	1.03	0.98 – 1.08	0.314	1.08	0.98 – 1.19	0.117	1.04	1.01 – 1.07	**0.012**	1.02	0.97 – 1.07	0.483	1.12	1.02 – 1.23	**0.013**
Temperature (10ºC increase)	1.02	1.00 – 1.03	**0.021**	1.01	0.99 – 1.04	0.256	1.02	0.97 – 1.07	0.424	1.00	0.98 – 1.02	0.770	1.00	0.96 – 1.04	0.977	1.01	0.95 – 1.08	0.785
RH (10% increase)	1.01	1.00 – 1.01	0.061	1.01	0.99 – 1.02	0.310	1.02	1.00 – 1.04	0.133	1.02	1.01 – 1.03	**0.001**	1.03	1.01 – 1.05	**0.001**	1.01	0.98 – 1.04	0.546
$PM_{2.5}$*AER	0.99	0.97 – 1.02	0.589	1.00	0.95 – 1.04	0.833	0.96	0.88 – 1.05	0.342	1.00	0.96 – 1.04	0.975	1.00	0.93 – 1.07	0.966	0.90	0.79 –1.03	0.133

Figure S.4. Estimated percentage change in cause-specific mortality associated with a 10µg/m³ increase in $PM_{2.5}$ at 15% and 75% AER, stratified by season, parcel housing and residential type, excluding both in-patient and outpatient in-hospital death, 2000-2015, Massachusetts, USA.

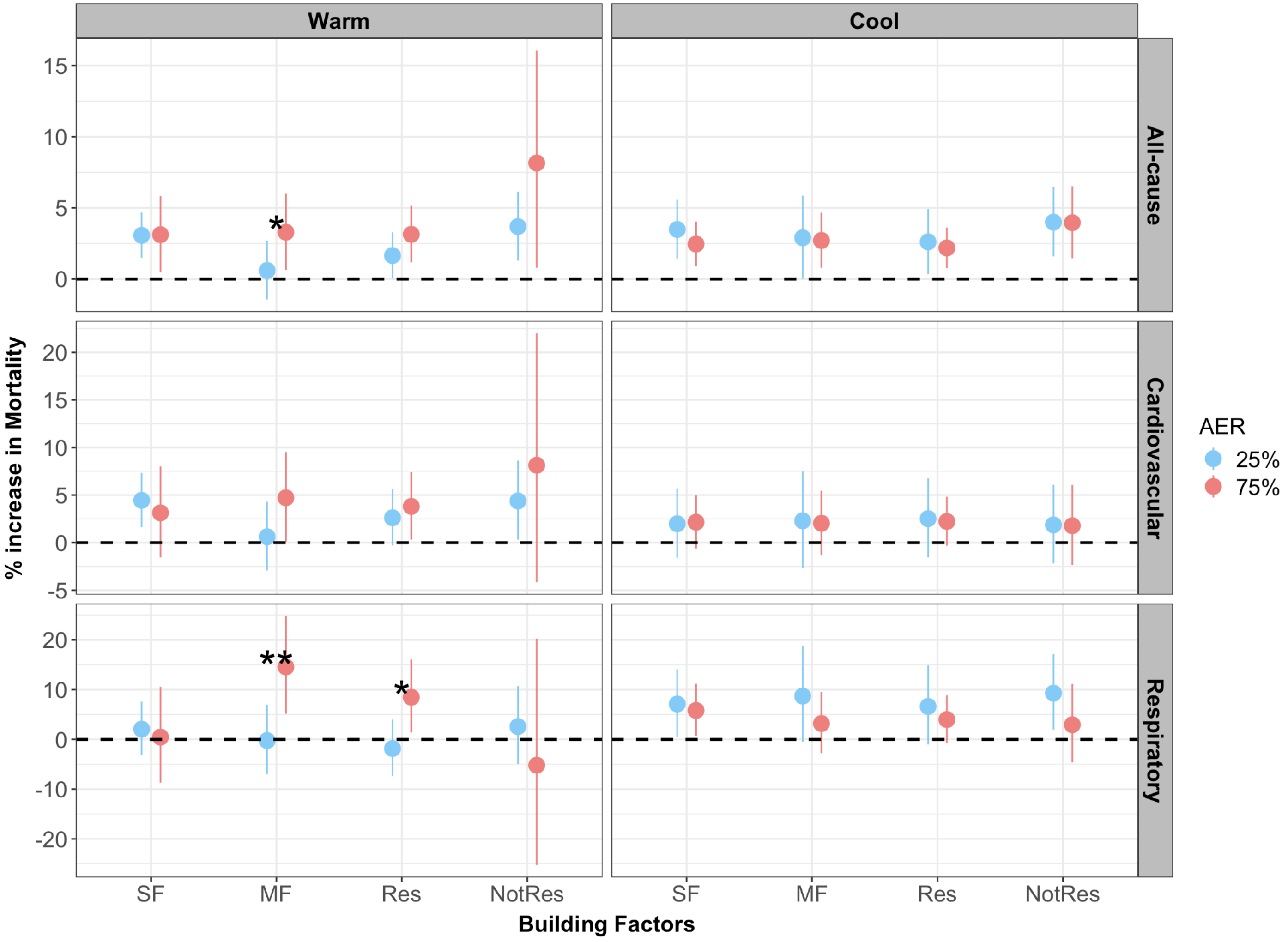

Note: Significance for the $PM_{2.5}$*AER product term on the multiplicative scale: * p<0.05; ** p<0.01;
Abbreviations: SF=Single Family; MF=Multifamily; Res=Residential; NotRes=Non-residential parcels
